# Selective Capture of Histidine-tagged Proteins from Cell Lysates Using TEM grids Modified with NTA-Graphene Oxide

**DOI:** 10.1038/srep32500

**Published:** 2016-10-17

**Authors:** Christopher J. Benjamin, Kyle J. Wright, Scott C. Bolton, Seok-Hee Hyun, Kyle Krynski, Mahima Grover, Guimei Yu, Fei Guo, Tamara L. Kinzer-Ursem, Wen Jiang, David H. Thompson

**Affiliations:** 1 Department of Chemistry, Purdue University, West Lafayette, Indiana 47907, USA; 2 Department of Biological Sciences, Purdue University, West Lafayette, Indiana 47907, USA; 3 Weldon School of Biomedical Engineering, Purdue University, West Lafayette, Indiana 47907, USA; 4 Center for Cancer Research, Purdue University, West Lafayette, Indiana 47907, USA

## Abstract

We report the fabrication of transmission electron microscopy (TEM) grids bearing graphene oxide (GO) sheets that have been modified with N^α^, N^α^-dicarboxymethyllysine (NTA) and deactivating agents to block non-selective binding between GO-NTA sheets and non-target proteins. The resulting GO-NTA-coated grids with these improved antifouling properties were then used to isolate His_6_-T7 bacteriophage and His_6_-GroEL directly from cell lysates. To demonstrate the utility and simplified workflow enabled by these grids, we performed cryo-electron microscopy (cryo-EM) of His_6_-GroEL obtained from clarified *E. coli* lysates. Single particle analysis produced a 3D map with a gold standard resolution of 8.1 Å. We infer from these findings that TEM grids modified with GO-NTA are a useful tool that reduces background and improves both the speed and simplicity of biological sample preparation for high-resolution structure elucidation by cryo-EM.

Single particle cryo-EM analysis (SPA) is a rapidly growing method for elucidating structure of biological materials at near atomic resolution^1^,^2^ due to recent advances in instrumentation and computational algorithms^3^. One aspect of the SPA process that is not well optimized, however, is sample preparation. Traditionally, proteins targeted for structural analysis must be overexpressed and subjected to time-consuming purification and concentration steps, sometimes under harsh conditions that disrupt protein-protein interactions of interest. Recently, there have been efforts reported that seek to address these limitations, either by improving grid rigidity to reduce beam-induced motion^4^,^5^,^6^ or by effecting on-grid purification with ‘affinity grids’^7^,^8^,^9^,^10^ that employ metal chelating lipids that were originally developed for two-dimensional protein crystallization at the lipid-water interface^11^,^12^,^13^,^14^. The latter approach seeks to selectively capture biological target molecules from complex mixtures such as cell lysates as an integral part of the TEM sample preparation process^10^,^15^.

Although lipid monolayer affinity grids have shown some success in producing samples for cryo-EM reconstruction at 20 Å resolution^7^, robust performance of the reported grid coatings may be limited by film instability and non-uniformity under the evaporative casting methods that are often employed. Additionally, these lipid films require a thin polymer layer or a holey carbon substrate layer to provide mechanical support of the deposited film. The electrical conductivity of monolayer graphene is six orders of magnitude higher than amorphous carbon, and although the level of conductivity in graphene decreases with the extent of oxidation, it has been shown to recover much of this conductivity upon reduction with H_2_ plasma^9^. Additionally, unlike unsupported lipid monolayers, the elasticity of graphene makes it ideal to resist permanent deformation due to mechanical transfer techniques from the material-water interface. Our interest in utilizing graphene-based affinity substrates is focused on exploiting the superior mechanical strength and conductivity it offers. By conferring better target specificity to this substrate, affinity graphenic substrates have the potential to offer both improved stability and resistance to non-specific adsorption such that direct capture from cell lysates may be feasible.

We sought to address the limitations of lipid monolayer coated affinity grids by employing a GO derivative that minimizes background signal due to the single atom thickness and improved conductivity as a way to combat sample charging and instability during image capture^16^. Here we demonstrate the utility of affinity grids using Langmuir-Schaefer (L-S) transfer of GO monolayer sheets that have been functionalized with covalently linked N^α^, N^α^-dicarboxymethyllysine (GO-NTA). Using these affinity grids, we were able to selectively capture both His_6_-T7 bacteriophage and His_6_-GroEL. When the prepared grids were further modified with bovine serum albumin (BSA), a common antifouling agent that limits non-specific adsorption of non-targeted cellular debris, we were able to selectively capture these proteins directly from bacterial lysate while avoiding deposition of non-target proteins (Fig. 1).

## Results and Discussion

### Synthesis of GO Sheets Functionalized with NTA

**Figure 2:**
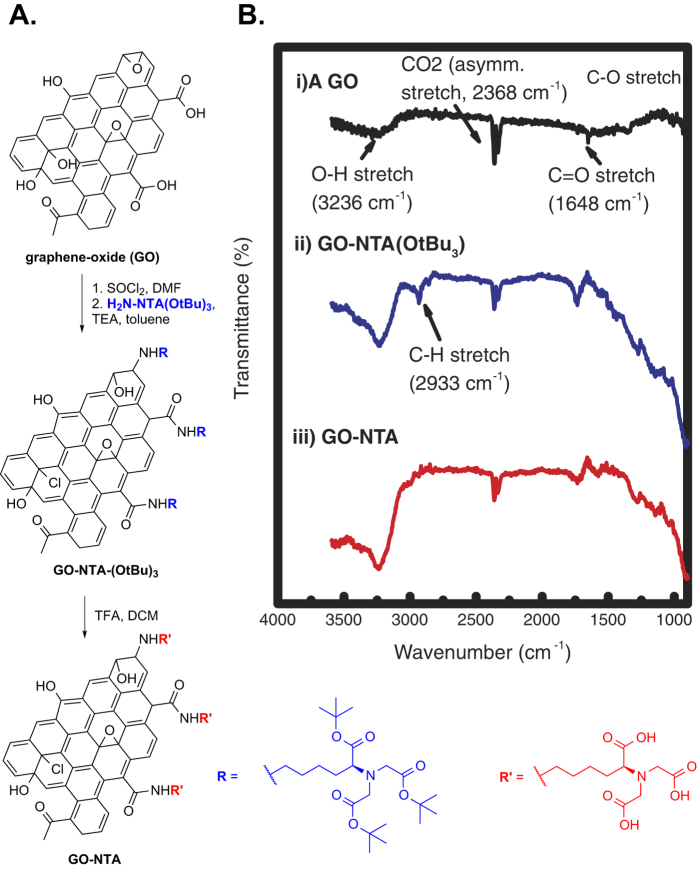
Synthesis and characterization of GO-NTA. (**A**) Reaction sequence for preparation of GO-NTA from GO. (**B**) FTIR spectra of (i) GO, (ii) GO-NTA (O-*t*-Bu)_3_, and (iii) GO-NTA.

GO was produced from graphene using Hummer’s method^16^. Activation of the GO carboxylic acid groups with SOCl_2_ prior to reaction with the tris-*t*-butyl ester of lysine NTA gave GO-NTA-(O-*t*-Bu)_3_. TFA deprotection of this intermediate gave GO-NTA (Fig. 2A). Fourier transfer infrared spectroscopy was used to monitor these reactions as shown in Fig. 2B 17. The spectra of GO displayed a broad absorption at 3236 cm^−1^ (O-H stretch) and a sharper absorption at 1648 cm^−1^ (C=O stretch)^16^. The NTA-GO tris-*t*-butyl ester displayed an additional absorption at 2933 cm^−1^ (C-H stretch) due to the incorporation of the lysine and *t*-butyl moieties. Following treatment of NTA-GO tris-*t*-butyl ester with TFA, the presence of the aliphatic C-H stretching was greatly reduced, indicating successful deprotection of the NTA chelator substituents^18^.

Previous work has shown that the typical GO sheet absorption band at ~240 nm is shifted to ~270 nm when the GO sheets are dispersed in aqueous solution. The origin of this hypsochromic shift is due to n-π* electronic transitions arising from the C=O bonds introduced by oxidation^19^. GO-NTA samples prepared in this manner exhibited a major absorption peak at ~280 nm (Figure S1), in good agreement with these reports.

### GO-NTA Monolayer Formation

Most reports of Langmuir dispersions of GO at the air-water interface focus on surfactant-assisted dispersion methods to stabilize GO sheets dispersed in water^20^. Treatment of GO with surfactants in these cases; however, biases the interfacial activity towards water-surfactant activity rather than GO-NTA activity due to their high relative abundance. The planar structure and functional group distribution on GO-NTA confers edge amphiphilicity due to the distribution of hydrophilic carboxyl, ketone, aldehyde, amide, and alcohol groups around the periphery of the hydrophobic aryl GO-NTA core (Fig. 2A)^21^. Since GO-NTA becomes increasingly hydrophobic as displacement toward the core from the GO-NTA edge increases, larger sheets will tend to be more hydrophobic and migrate to the air-water interface, whereas smaller more hydrophilic GO-NTA sheets are displaced into the aqueous subphase by the larger GO-NTA sheets^22^.

Previously, it was thought that the use of surface-active agents was needed to disperse GO sheets. More recent work; however, has shown that GO layers form at the air-water interface in the absence of surfactant molecules^23^. Brewster angle microscopy studies have shown that the interfacial refractive index of GO solutions change after a few hours of stirring pure GO dispersions in water^24^. This suggests a time-dependent mechanism for GO absorption at the air-water interface, with slower interfacial adsorption rates attributable to slower diffusion rates of large graphene sheets relative to typical surfactant molecules used for studies of the air-water interfaces. To increase the amount of surface-active graphene sheets present at the air-water interface while circumventing hours of stirring, studies using rising gas bubbles of CO_2_ and N_2_ as a way of transporting these sheets to the surface have proven successful^22^. These studies show that GO can migrate to the air-water interface, while other work suggests that the surface activity of GO sheets in water can be increased using volatile, polar protic solvents to enable their manipulation using Langmuir compression^23^.

**Figure 3:**
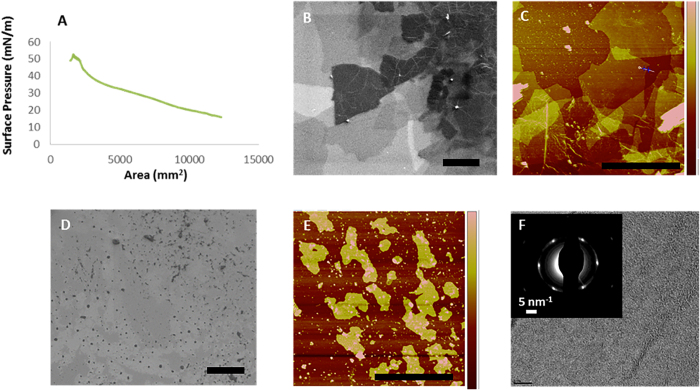
Characterization of GO-NTA surfaces. (**A**) Pressure-area isotherm for GO-NTA sheets at the air-water interface, dispersed at 67 ng/mL in water at 20 °C. GO-NTA sheets compressed at a rate of 500 mm^2^/min. (**B**) SEM images taken 1.0 keV, with 5 μm scale bar and (**C**) AFM images of GO-NTA after LS-transfer onto Si wafers from a subphase of pure H_2_O (5 μm scale bar). (**D**) SEM images taken at 0.5 keV (5 μm scale bar) and (**E**) AFM of GO-NTA after LS-transfer onto Si wafers from a subphase of IPA/H_2_O (5 μm scale bar). (**F**) TEM image of GO-NTA monolayers after L-S transfer from a subphase of IPA/H_2_O onto TEM grids; Inset: Selected area electron diffraction analysis of GO-NTA monolayer.

Compression of the GO-NTA material at the interface gave a characteristic surface pressure-area isotherm (Fig. 3A), suggesting a progression from isolated GO-NTA sheets to close edge-to-edge packing of GO-NTA sheets, followed by folding, wrinkling, and sliding of the nearest neighbor GO-NTA sheets atop one another upon further compression^25^, in a manner analogous to pressure-induced collapse of Langmuir phospholipid monolayer films^23^. Repulsive electrostatic interactions and attractive van der Waals forces compete as GO-NTA sheets come into close contact. Previous work with GO monolayers has suggested that over-compression of GO causes irreversible coagulation above ~15 mN/m^23^ due to the increasing participation of attractive van der Waals interactions once the repulsive electrostatic interactions between sheet edges has been overcome by lateral compression. Transfer of these films onto silicon substrates at multiple surface pressures enabled the transfer of single layer GO sheets at surface pressures above 15 mN/m.

### Characterization of GO-NTA Monolayers

Epifluorescence microscopy, AFM, and SEM was employed to determine the thickness and lateral distribution of GO-NTA sheets deposited onto solid substrates by L-S transfer from the air-water interface^26^. In particular, epifluorescence microscopy of F-PABA-GO-NTA monolayers proved useful because opacity of the graphene-based sheet is directly related to its thickness as revealed by analysis of monolayer-coated grid and negative control bare Cu TEM grid samples that showed significantly greater fluorescence intensity for grids coated with F-PABA-GO-NTA (Figure S2).

We then evaluated drop casting and L-S transfer deposition methods for the production of the thinnest films possible, while yielding films with the highest density of NTA capture ligands. Drop casting^26^,^27^, followed by slow evaporation of solvent, resulted in a fluorescence signal that completely spanned the holes of Formvar-coated 400 mesh Cu grids (data not shown). Slow solvent evaporation enables GO sheets to settle on top of one another to form a multi-layered film covering the TEM grid holes. Although successful, our findings suggest that drop casting typically yields sheets that are too thick and heterogeneous for protein structure elucidation applications by cryoEM. L-S transfer in the presence of 2-propanol (IPA) proved successful for depositions onto 1500 mesh grids, both with and without Formvar coating; however, L-S transfers with pure water resulted in thicker heterogeneous coatings over a limited area of the holes. L-S transfer onto Si wafers under identical conditions confirmed the presence of multilayered films (Fig. 3B,D). We were not able to fabricate functional coatings with 400 mesh grids using L-S transfer, a finding we attribute to the mismatch between the average GO-NTA sheet size of ~16 μm × 16 μm and the 37 μm × 37 μm grid holes of these grids. It is worth noting that the GO-NTA sheet size can vary as a function of oxidation reaction duration and sonication time employed during GO synthesis^28^.

To gain further insight into the structure of these GO-NTA films, SEM and AFM analyses were performed after compression to 15 mN/m and L-S transfer of GO-NTA monolayer sheets onto Si wafers. To prepare Si wafers for L-S transfer, ~2.25 cm^2^ wafers were cut and glued (bottom side) onto a transfer tube. The surface pressure was maintained until the Si wafer contacted the monolayer; the film was then recompressed to 15 mN/m after the L-S transfer step. The area difference before and after L-S transfer indicated transfer efficiencies of 75–85%. Image analysis of the coated Si wafers revealed the presence of GO-NTA monolayer sheets transferred from IPA-containing subphases with ~1.3 nm thicknesses that were relatively uniform (Fig. 3D,E), in good agreement with previously reported values for single layer GO^29^. In the absence of IPA; however, data from SEM and AFM experiments revealed GO-NTA films comprised of overlapping sheets and undesirable layer thickness variations (Fig. 3B,C).

Selected area electron diffraction analysis of GO-NTA L-S films, deposited onto bare 2000 mesh grids from the air-IPA/H_2_O interface, revealed a hexagonal diffraction pattern, indicative of a single layer of graphenic material (Fig. 3F). The measured intensity of the inner and outer peaks confirms the presence of a single GO-NTA layer (Figure S3)^27^.

### Affinity Capture of His_6_-T7 Bacteriophage from *E. coli* Lysate Using GO-NTA Monolayer Purification and PABA + BSA as Antifouling Agents

**Figure 4:**
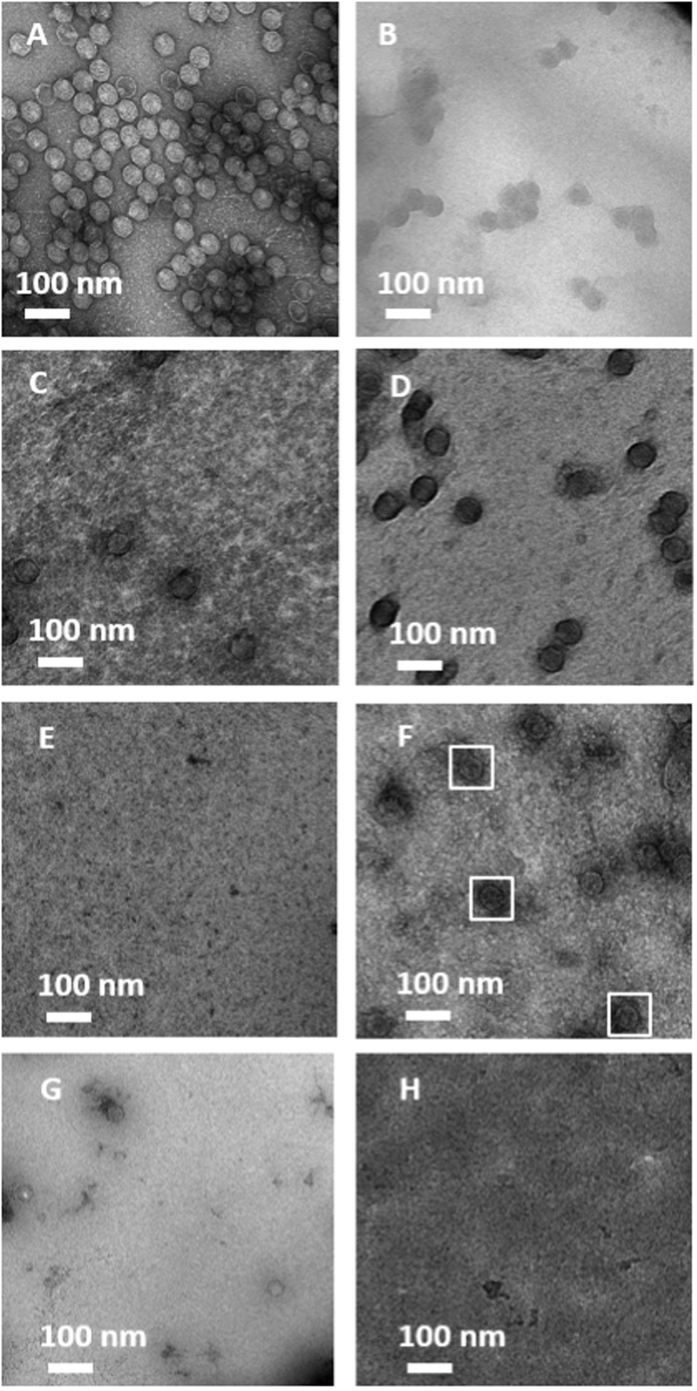
Micrographs of negatively stained his_6_-T7 bacteriophage using various TEM grid coatings: (**A,B**) GO-NTA; (**C,D**) PABA-GO-NTA; and (**E–H**) BSA-PABA-GO-NTA. Negative controls (**A,C,E**) demonstrate no capture of purified phage when Ni^2+^ is absent, whereas coatings treated with Ni^2+^ (**B,D,F**) show capture of purified phage. Affinity capture of phage from lysate (**G**) can be reversed by incubation of (**G**) with 500 mM imidazole (**H**) that removes the Ni^2+^ from the coating and abrogates the affinity interaction between the phage and the grid surface.

The capacity of GO-NTA coated grids to capture His_6_-T7 bacteriophage (His_6_-T7) by affinity interaction was examined first by negative-stain TEM. After a 2 min exposure of purified His_6_-T7 on GO-NTA modified 1500 mesh grids, dense clusters of phage particles were found on the GO-NTA surface in the absence of Ni^2+^ (Fig. 4A). Paradoxically, we observed fewer phage particles after charging the GO-NTA grids with Ni^2+^ (Fig. 4B). We attribute these findings to non-specific and random covalent coupling of lysine residues with epoxide and aldehyde residues on the GO sheets that are inactivated upon exposure to the metal ion^30^. To obviate this problem, we chemically deactivated these functional groups by treatment of GO-NTA with 4-aminobenzoic acid (PABA) after L-S transfer. The resulting PABA-GO-NTA grids showed a reduction in, but incomplete abrogation of, non-specific His_6_-T7 binding under the same incubation conditions (Fig. 4C). When activated with Ni^2+^, PABA-GO-NTA grids revealed a higher density of phage particles due to engagement of the NTA:Ni^2+^:His_6_ affinity interaction (Fig. 4D). To further enhance the anti-fouling properties of this material, we incubated the PABA-GO-NTA grids with BSA immediately before the affinity capture step. Under these conditions, BSA appears to complete the blockade of non-specific viral particle adsorption (Fig. 4E), suggesting that BSA inhibits non-specific binding more effectively than PABA modification. After Ni^2+^ activation of the BSA-blocked PABA-GO-NTA surfaces, we observed a recovery in His_6_-T7 binding to the grids (Fig. 4F). To further demonstrate the Ni^2+^ dependence of this interaction, we treated the grid with 500 mM imidazole, leading to the elution of His_6_-T7 from the grid (data not shown). Taken together, these findings demonstrate the importance of deactivating highly reactive chemical functionalities on the surface of GO prior to use in affinity capture experiments.

Next, we sought to capture His_6_-T7 directly from clarified *E. coli* lysate. The engineered His-tag does not interfere with His_6_-T7 infectivity, thereby enabling the infection of BL21 cells and viral replication *in vitro*. A negative control experiment demonstrated that Ni^2+^-free BSA-PABA-GO-NTA grids resulted in little or no capture of phage and minimal background adsorption from non-targeted cellular material (Figure S4); however, Ni^2+^ activation prompted selective His_6_-mediated binding of bacteriophage to the grid surface (Fig. 4G). As an additional control, the grid was washed with 500 mM imidazole after Ni^2+^, but prior to incubation with lysate, to demonstrate that imidazole stripping of the metal ion results in the abrogation of His_6_-T7 binding (Fig. 4H). These results indicate that BSA-PABA-GO-NTA coated grids are able to effectively purify His_6_-T7 directly from clarified lysate on the grid using the reversible NTA:Ni^2+^:His_6_ affinity interaction.

### Affinity Capture of GroEL From *E. coli* Lysate Using BSA-PABA-GO-NTA Monolayer Purification

**Figure 5:**
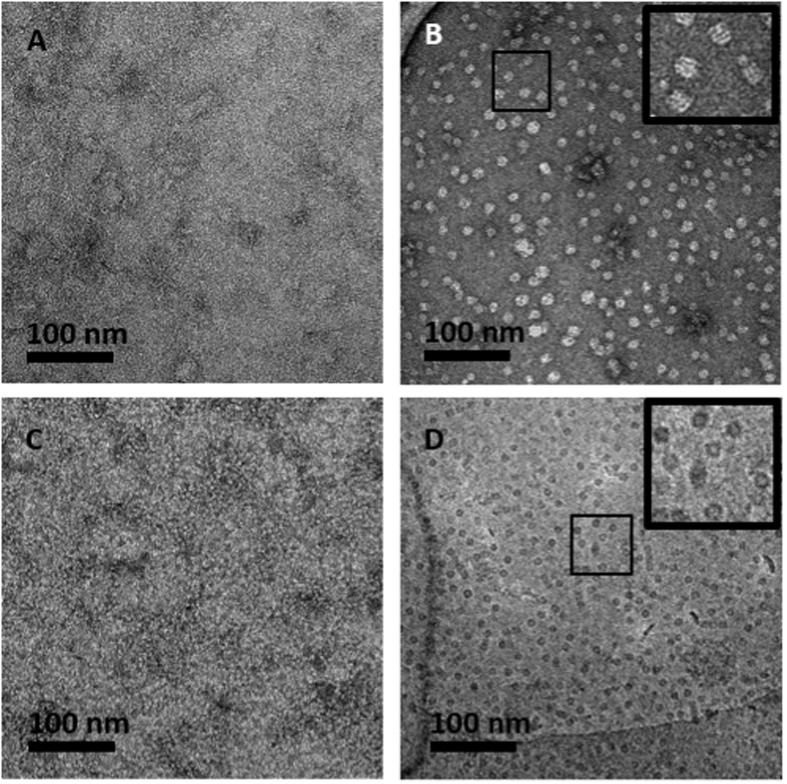
Micrographs of his_6_-GroEL lysate affinity capture using BSA-PABA-GO-NTA TEM grid coating. Micrographs (**A–C**) are negatively stained. (**A**) Negative control showing no capture of his_6_-GroEL when Ni^2+^ is absent. (**B**) Affinity coating activated with Ni^2+^ displays specific capture of his_6_-GroEL from lysate. Treatment of the grid in (**B**) with 500 mM imidazole (**C**) leads to Ni^2+^ stripping from the coating and abrogation of his_6_-GroEL capture. (**D**) Representative cryo-EM image of affinity captured his_6_-GroEL from lysate.

The performance of antifouling BSA-PABA-GO-NTA coatings for high-resolution single particle reconstruction analysis was then evaluated by performing on-grid affinity capture of His_6_-GroEL from *E. coli* lysates. As observed for His_6_-T7 capture, specific binding of His_6_-GroEL occurred only with Ni^2+^-activated (Fig. 5B), but not Ni^2+^-free (Fig. 5A) or 500 mM imidazole treated grids (Fig. 5C). Next, we obtained cryo-EM images of His_6_-GroEL deposited onto BSA-PABA-GO-NTA coated grids (Fig. 5D). Initial attempts at His_6_-GroEL capture and cryofixation on 1500 mesh grids coated with BSA-PABA-GO-NTA generated unacceptably thick sample vitrification; however, high quality samples of His_6_-GroEL captured from lysate were afforded by BSA-PABA-GO-NTA films deposited by L-S transfer onto lacey carbon-supported 400 mesh copper grids.

### Single Particle Analysis of His_6_-GroEL

**Figure 6:**
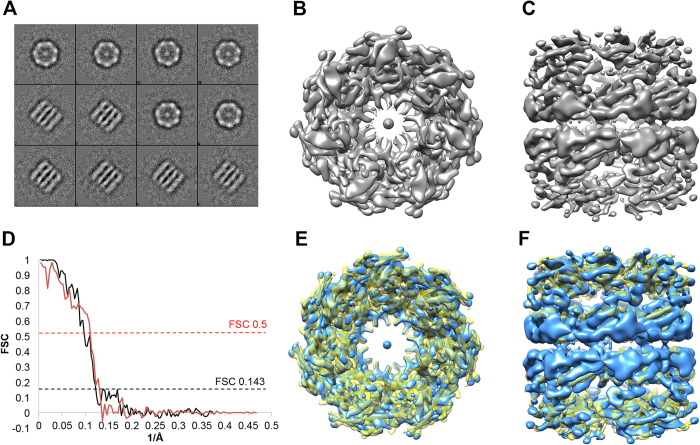
SPA of his6-GroEL captured from lysate. (**A**) Class averages of his_6_-GroEL images captured from BSA-PABA-GO-NTA coated grids that were used to build the initial model; (**B**) Top and (**C**) side views of refined his_6_-GroEL EM map at 8.1 Å resolution (gold standard, 0.143 criteria); (**D**) Fourier Shell Correlations: gold standard using conservative masking (black), and cross-validation between published GroEL map EMD-5001 and our map (red); (**E**) Top and (**F**) side views of overlay with our his_6_-GroEL EM map (blue) and EMD-5001 (yellow)^34^.

EMAN 2.1^31^ was used for single particle analysis of His_6_-GroEL deposited onto BSA-PABA-GO-NTA coated grids since this protein target is often used for gauging workflow performance and data processing robustness^32^,^33^,^34^. The reconstruction effort followed the usual steps from within the application, except that the particles were manually picked. Background signal contributions by the BSA blocking layer may have contributed to the difficulties encountered during attempts at automated particle picking. Nonetheless, 5363 particles were hand-picked from 217 micrographs and the particles rapidly converged into coherent classes displaying high contrast (Fig. 6A).

Of 50 total class averages, 12 were chosen to produce an initial model with imposed D7 symmetry (Fig. 6A). After 12 refinement iterations with an angular sampling of 1.76 degrees, we were able to produce a gold standard (0.143 criteria) density map having 8.1 Å resolution using conservative masking (Fig. 6B–D). There were visible nodes in the FSC curve at regular intervals that resulted from an uneven distribution of micrograph defocuses.

To probe the accuracy of our model, we performed a comparison with published 4.2 Å resolution cryo-EM map EMD-5001^34^ that was also produced by EMAN using D7 symmetry. Chimera^35^ was used to fit the volumes before calculating the FSC, yielding a 9 Å resolution using 0.5 criteria (Fig. 6D–F).

A substantial difference between our map and the published structure was observed, wherein additional electron density within the inner pore of the protein was found in the case of our His_6_-GroEL map. We attribute this finding to the extended amino acid sequences at the N- and C-termini (i.e., MRGSHHHHHHTDPALRA and GLCGR, respectively) of our His_6_-GroEL construct derived from the ASKA Library.

Wild type N- and C-termini of the protomers are located at the surface of the inner pore lining the assembled tetradecameric complex. Thus, the 14 engineered subunits comprising our His_6_-GroEL complex yield an additional 308 residues that occupy the pore, of which 84 are histidines. Given the large number of potential metal chelation regioisomers and topoisomers, as well as the high potential for conformational flexibility in the N- and C-terminal sequences, we believe that this density is unlikely to adopt a defined structure and instead appears as a filled “droplet” within each ring. Also, there is a noticeable decrease in density in the flexible apical region that suggests less structural coherency. We infer from these findings that the additional pore residues, along with NTA chelation, may create dynamic distortions to the structure of GroEL that could vary for each particle, reducing coherency and map density at the apical ends. Further experiments that vary the length and number of his-tag linkers per particle may allow for a better model convergence with improved resolution, and potentially improved resemblance to wild type GroEL.

## Conclusions

Our findings show that a new functionalized GO-NTA monolayer sheet can be used as a coating to facilitate on-grid affinity purification from clarified cell lysates for negative stain TEM and cryo-EM single particle reconstruction analysis. GO sheets were successfully functionalized with lysine-NTA affinity ligands and compressed monolayer films at the air-water interface were prepared by employing an IPA/H_2_O mixture to lower surface tension before L-S transfer onto EM grids. SEM, AFM, fluorescence spectroscopy, and TEM analysis of these films suggests that single monolayer sheets of GO-NTA can be transferred onto Si wafers, bare copper grids, and holey carbon grids using this method. Since GO films are thinner than amorphous carbon substrates and offer better electron dissipation than lipid-based affinity grids, we believe these two benefits will yield improved contrast for cryo-EM image acquisition of biological molecules.

Two blocking techniques, PABA coupling and BSA adsorption, were needed to deactivate the reactive GO surface towards non-specific adsorption of occult impurities present in complex samples such as cell lysates. Our data shows that further investigations into blocking agents that minimize background noise and improve cryofixation reliability are needed. Nonetheless, this grid coating approach showed good specificity for capture of His-tagged T7 bacteriophage and GroEL from highly complex cell lysates, while limiting background adsorption of non-targeted cellular material.

The utility of these grids for on-grid purification from cell lysates and single particle reconstruction was demonstrated using His_6_-GroEL. Capture of this target onto the surface of GO-NTA affinity grids from clarified cell lysates was then used as the key step to enable selection of 5363 particles for reconstruction analysis, yielding a map with a gold standard resolution of 8.1 Å. The presence of additional His-tag and linker amino acids in our engineered GroEL was distinctly visible in our density map, but our final map could still be fit to a published high-resolution EM map to 9 Å resolution using 0.5 criteria. We conclude based on these findings that affinity capture-based graphenic materials offer great potential for simplified and accelerated cryo-EM sample preparation for high-resolution structure elucidation. These coatings possess many advantages over NTA-lipid-modified grids^7^,^8^ or other grid coatings that lack protection from non-specific binding^4^,^5^,^6^, thereby offering substantial improvements in sample preparation time and reduced sample volumes needed to acquire high resolution structural information of a given protein target and, potentially, its interaction partners.

## Experimental Methods

### Graphene-Oxide-NTA Synthesis

GO was synthesized as described by Marcano *et al*.^16^. This intermediate (335 mg) was stirred in a mixture of SOCl_2_ (60 mL) and DMF (1.5 mL) at 70 °C for 3 d before evaporating the SOCl_2_ and DMF and washing the residue with dry DCM (3 × 50 mL). ACN (50 mL) and Et_3_N (3 mL) were then added and the mixture stirred for 30 min. Tris(O-*t*-butyl)-N^α^,N^α^-dicarboxymethyllysine ester (533 mg) was then added and the mixture stirred at 100 °C for 3 d before washing with THF and H_2_O (9,000 rpm for 15 min, 3 times for each solvent), and vacuum drying at 60 °C for 24 h. TFA (10 mL) in THF (30 mL) was added to the dried *t*-butyl-NTA ester intermediate (180 mg) and stirred at 60 °C for 5 h before washing with THF and H_2_O (11,000 rpm × 15 min, 3 times for each solvent)^29^,^36^.

### GO-NTA Exfoliation

The GO-NTA sheets were ultrasonically exfoliated at 1 mg/mL by suspension of the powder in 5:1 MeOH:H_2_O using probe sonication at 150 watts for five cycles (45 s sonication followed by 45 s of rest in each cycle). The product was centrifuged at 1200 g for 10 min, after which the supernatant of exfoliated GO-NTA sheets was removed from the sediment of aggregated sheets and subjected to another 5 rounds of sonication. A final centrifugation at 1200 g for 10 min was performed prior to removal of the supernatant to yield a GO-NTA solution that was stored for subsequent grid coating experiments.

### Langmuir-Trough Setup

Exfoliated GO-NTA was deposited at the air-water interface of a Kibron μTrough via a syringe pump fitted with a 20 mL syringe. The GO-NTA dispersion was loaded into the syringe and slowly introduced at the air-water interface at a rate of 100 μL/min until the surface pressure reached 15 mN/m. The film was then allowed to relax for 5 min, followed by slow compression of the film to 15 mN/m. IPA was then added to the subphase and the film transferred to either Si wafers, bare 1500 mesh TEM grids, or holey carbon grids by Langmuir-Schaefer (L-S) transfer.

### 4-Aminobenzoic acid (PABA) Modification of GO-NTA

GO-NTA (1 mg/mL) was partially deactivated by adding PABA (30 mg) to a 10 mL GO-NTA dispersion. This mixture was probe sonicated at 150 W for 30 sec of continuous sonication, followed by shaking for 24 h on a rotary mixer. The PABA-GO-NTA was then exfoliated and washed as described above for GO-NTA exfoliation.

### Fluorescein Modification

Fluorescein modification of GO-NTA was performed by adding 2 mg of aminofluorescein to an aqueous solution of PABA-GO-NTA (10 mL at 1 mg/mL). This mixture was probe sonicated for 30 s at 150 W of continuous sonication and then placed on a rotary mixer in the dark for another 24 h. The material was then centrifuged to pellet the GO species before re-suspending in water, addition of 5:1 MeOH:H_2_O, re-pelleted, and decanted a total of 10 times before exfoliation of the Fluorescein-PABA-GO-NTA (F-PABA-GO-NTA) product as described above for GO-NTA.

### Bovine Serum Albumin (BSA) Modification

Following L-S transfer of GO-NTA or PABA-GO-NTA onto EM grids and overnight drying in a desiccator, the grids were placed on a strip of Teflon before addition of BSA (10 μL of 0.1 mg/mL) and incubation for 5 min, followed by 3 × 20 μL double deionized H_2_O washes. The modified grids were then stored in a desiccator until use.

### Fluorescence Microscopy Sample Preparation

F-PABA-GO-NTA was deposited onto 1500 mesh grids in the dark by L-S transfer as described above. After transfer, the grids were allowed to dry in the dark for 1 d before sandwiching them between a glass and cover slip with 5 μL of double deionized H_2_O and the sandwich sealed with nail polish. The glass slide was then mounted on a light microscope for epifluorescence imaging.

### GO Concentration Measurements

The concentrations of the GO-NTA dispersions were measured at different steps of the synthesis by monitoring the UV-vis spectra of the products. The extinction coefficient data used for one batch of GO-NTA is shown (Fig. S1A). Since each batch of GO-NTA has minor differences in concentration, each preparation was evaluated for its own experimentally determined extinction coefficient for subsequent concentration measurements. Standard solutions used to determine the extinction coefficients were prepared by dispersing a weighed amount of dry GO-NTA into known volumes of 5:1 MeOH:H_2_O and measuring the absorbance at 280 nm across a series of dilutions with 5:1 MeOH:H_2_O. The extinction coefficient was derived from the slope of these concentration-dependent absorption plots.

### GO-NTA Grid Treatment with Purified His_6_-T7 Bacteriophage

Purified C-terminal gp10 His_6_-T7 bacteriophage was initially prepared at a concentration of 10^12^ particles/mL, with dilution to 10^10^ particles/mL in HEPES buffer (pH = 7.4), before application to the affinity grid surface. GO-NTA modified grids were placed on a Teflon strip, 1 mM NiSO_4_ (10 μL) added, and the grids incubated for 5 min before washing with double deionized H_2_O (2 × 20 μL) and HEPES buffer (1 × 20 μL). Purified phage (3.5 μL) was then applied to the surface and incubated for 2 min before washing with HEPES (2 × 20 μL), double deionized H_2_O (1 × 20 μL), and staining with 2% uranyl acetate (5 μL).

### GO-NTA Grid Treatment with His_6_-T7 Bacteriophage Lysate

BL21 bacterial cells in 1 mL of LB media were grown to an OD of 0.8 before adding 1.0 μL of His_6_-T7 bacteriophage (1 × 10^12^ particles/mL) to the media and shaking the culture for 1 h. After bench top centrifugation of the cells, the supernatant was withdrawn for use in His_6_-T7 bacteriophage particle capture studies. The grids were Ni^2+^-activated as described above, except that His_6_-T7 lysate (5 μL) was applied to the surface before incubation for 2 min. The grids were then washed with HEPES (2 × 20 μL), double deionized H_2_O (1 × 20 μL), and then stained with 2% uranyl acetate (5 μL).

### GO-NTA Grid Treatment with His_6_-GroEL Lysate

The ASKA Library was used to express N-terminal His_6_-GroEL. Cells containing N-His_6_-GroEL gene overexpression vector were grown to OD = 0.6 (in 100 mL of LB broth using a 37 °C shaker/incubator) and induced with a final concentration of 1.0 mM IPTG, before allowing the cells to grow for an additional 4 h. After centrifugation and removal of the supernatant, the cell pellet was re-suspended in lysis buffer (20 mM HEPES, 100 mM NaCl, pH = 7.4, 100 μg aprotinin, 174 μg phenylmethanesulfonyl fluoride (PMSF), and 500 μg of lysozyme) and allowed to sit for 20 min. Further disruption of the cell membranes was effected by 110 W probe sonication (35 pulses at 1 second/pulse), followed by centrifugation at 11,000 g for 10 min. The supernatant containing His_6_-GroEL was diluted 10-fold and assayed for affinity binding using the Ni^2+^-activation procedure described above, except that N-His_6_-GroEL lysate (5 μL) was applied to the surface and incubated for 2 min. The grids were then washed and stained with 2% uranyl acetate as described above.

### Affinity Capture of His_6_-GroEL from *E. coli* Lysates onto BSA-PABA-GO-NTA Grids for Cryo-EM Imaging

Samples were prepared as described above for negative stain TEM imaging, except that BSA-PABA-GO-NTA modified grids were exposed to His_6_-GroEL lysate, after which the excess solution was removed by blotting twice for 6 s per blot using an offset setting of −1 at 80% humidity using a Vitrobot device (FEI Company). The grids were then plunged into liquid ethane for cryofixation and imaged at 300 kV on an FEI Titan Krios equipped with a Gatan K2 Summit direct electron detection camera using low-dose techniques. Integrated microscope automation software (Leginon)^37^ was used to acquire a large set of micrographs at 11,000x magnification with an exposure time of 7.6 sec.

### Single Particle Analysis of His_6_- GroEL

Direct electron detector movie frames were processed in Appion^38^ to produce a set of averaged, motion-compensated micrographs to be used in subsequent steps. The micrographs had a 1.32 Å^2^/pixel resolution across a 4096 Å × 4096 Å array. EMAN 2.1 software^31^ was used for reconstruction of 5363 particles that were manually picked from 217 micrographs using a box size of 256. Automatic contrast transfer function (CTF) estimation and structure factor were determined from the incoherent sum of particles using e2ctf and phase-flipped to generate high-pass CTF-corrected particle stacks. Defocus was estimated to range between 0.4 μm–4 μm, but 55% of the particles were defocused between 2 μm–3 μm which resulted in a somewhat jagged CTF slope. Particles were binned 2X for class averaging and 12 classes were chosen to create an initial model with imposed D7 symmetry. The classes contained a mix of top and side views. In the refinement steps, the input set of particles was divided into even/odd halves, each containing 2682 particles. Two independent refinements were generated, producing a gold standard of 8.1 Å (using 0.143 criteria) after 12 iterations over two refinements with an angular sampling of 1.76 degrees. Additionally, we performed Fourier shell correlation against an existing high-resolution cryo-EM map, EMD-5001^34^. The maps were rotated and translated using Chimera^35^ to fit the volumes together. The correlation of our model against EMD-5001 (4.2 Å) gave an approximate resolution of 9 Å.

## Additional Information

**How to cite this article**: Benjamin, C. J. *et al*. Selective Capture of Histidine-tagged Proteins from Cell Lysates Using TEM grids Modified with NTA-Graphene Oxide. *Sci. Rep.* **6**, 32500; doi: 10.1038/srep32500 (2016).

## Supplementary Material

Supplementary Information

## Figures and Tables

**Figure 1:**
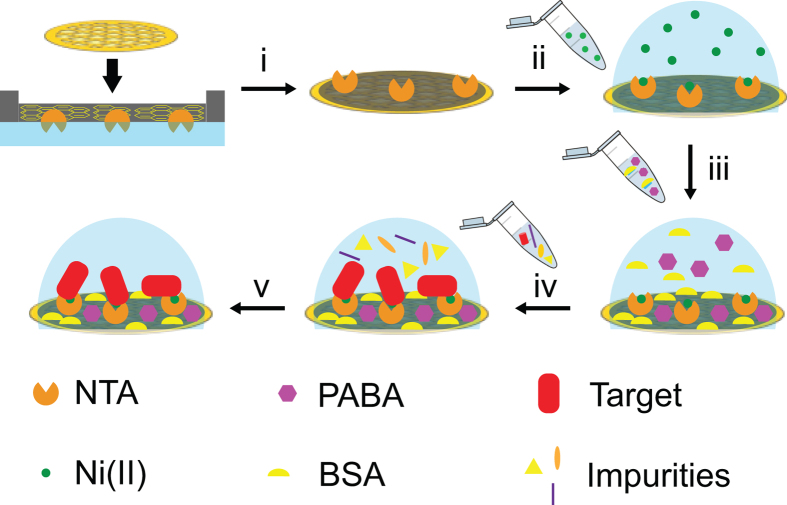
Conceptual diagram of sample preparation using a GO-NTA modified TEM grid. (i) GO-NTA monolayer deposition onto TEM grid via L-S transfer; (ii) activation of NTA with Ni^2+^; (iii) blocking of non-specific reaction and/or adsorption sites with 4-aminobenzoic acid (PABA) and bovine serum albumin (BSA); (iv) incubation of clarified lysate with blocked grid; (v) washing of non-target molecules from grid, followed by cryo-fixation or staining.
