# Supplementary material for: Selective Capture of Histidine-tagged Proteins from Cell Lysates Using TEM grids Modified with NTA-Graphene Oxide
Source: Sci Rep. 2016 Oct 17;6:32500. doi: 10.1038/srep32500 (PMC5066248; doi:10.1038/srep32500)
Supplement: Supplementary Information [file srep32500-s1.pdf]

Supplemental Information

Selective capture of histidine-tagged proteins from cell lysates using TEM grids modified with NTA-graphene oxide

Christopher J. Benjamin[a,1], Kyle J. Wright[a,1], Scott C. Bolton[a,1], Seok-Hee Hyun[a], Kyle Krynski[a], Mahima Grover[a], Guimei Yu[b], Fei Guo[b], Tamara L. Kinzer-Ursem[c], Wen Jiang[b], David H. Thompson[a,c]

Graphene-Oxide-NTA Synthesis. GO was synthesized using an improved Hummers' method that is easier to execute, is higher yielding, and does not evolve toxic gases (Figure 1B). It has been reported that there is no decrease in conductivity in the final product between the original and improved method, making it an attractive route for large scale production of GO [Marcano, D.C., *et al. ACS Nano* **2010** *4*, 4806-4814]. When a 9:1 mixture of H_2SO_4 and H_3PO_4 (130 mL total volume) was stirred with 1 g of graphite flakes (F516 flake graphite, 200-300 mesh, Asbury Carbons, Inc.) and $KMnO_4$ (6.0 g, 6.0 wt. equiv.), the reaction began with heating to ~40 °C and proceeded with further heating and stirring at 50 °C for 12 hours before cooling to 20 °C and pouring the reaction mixture into 120 mL of ice cold-water with 1 mL 30% H_2O_2. Next, this suspension was passed through a metal U.S. Standard testing sieve (W.S. Tyler, 300 µm) and then passed through a glass wool plug to filter larger particulates. The filtrate was then centrifuged at 4,000 rpm for 4 h, the supernatant discarded, and the pellet washed twice with a 1:1:1 volumetric ratio of H_2O, 30% HCl, and EtOH before passing the material through the testing sieve and centrifuging the filtrate at 4,000 rpm for 4 h to pellet the aggregated material. The supernatant was precipitated with Et_2O (200 mL) and filtered through a 0.45 µm PTFE membrane to gather the solid. The final material was dried under a 15 µm vacuum for 12 h, yielding 1.8 g of GO.

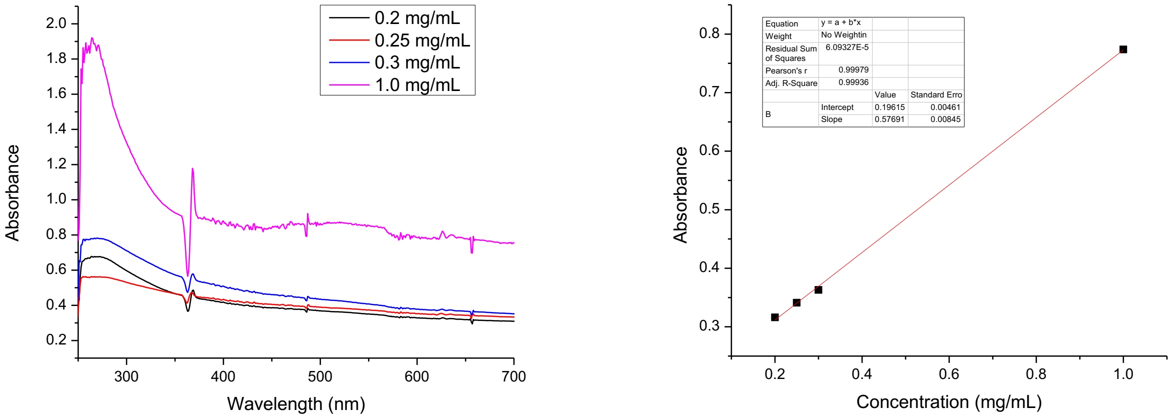

Figure S1. A: Absorption spectra for GO-NTA as a function of concentration; and B: Calibration curve for GO-NTA.

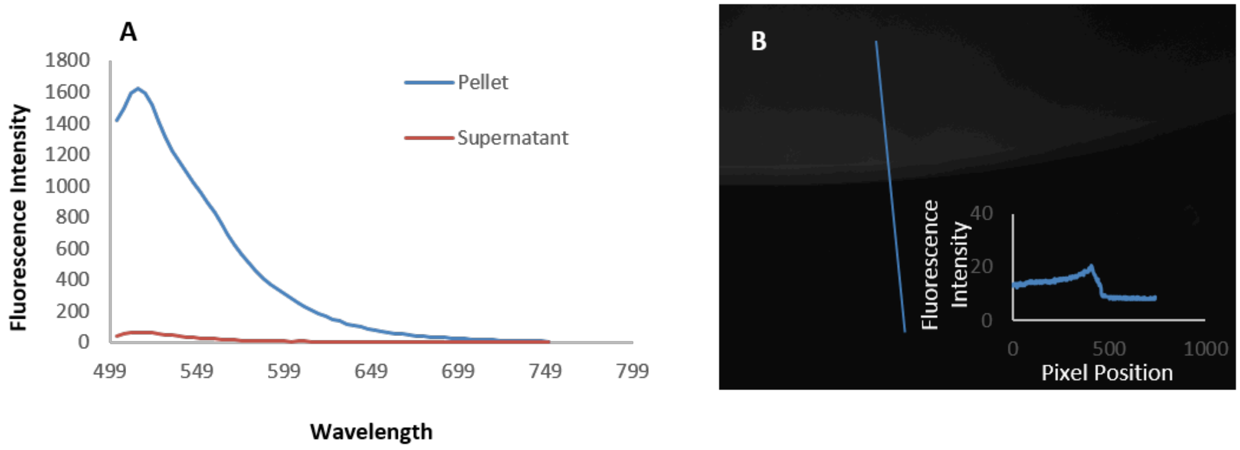

Figure S2. Analysis of Fluorescein-PABA-GO-NTA films by (A) fluorescence spectroscopy and (B) epifluorescence microscopy. Fluorescence spectra were measured for the supernatant (A, red spectrum) and the pellet (A, blue spectrum) after reaction of PABA-GO with amino-fluorescein. Epifluorescence image of F-PABA-GO-NA after LS-transfer onto a 400 mesh TEM grid; Inset: position-dependent intensity of F-PABA-GO-NA film on the TEM grid across the region indicated by the line in the epifluorescence image.

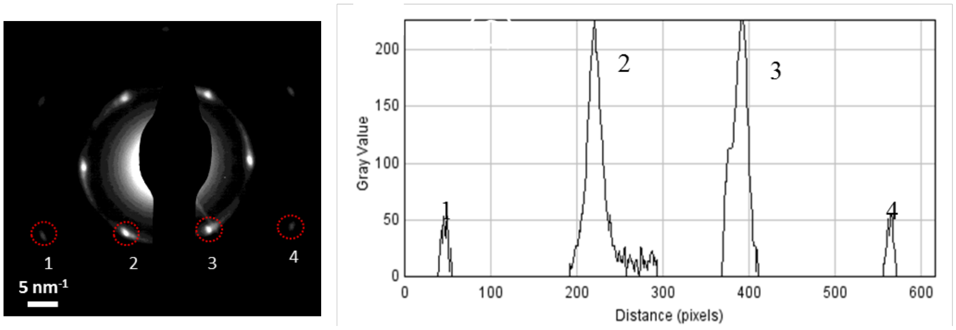

Figure S3. Selected area electron diffraction analysis of GO-NTA film on TEM grid. The hexagonal pattern of sharp diffraction peaks (left), as well as their spacing and intensity (right) are consistent with deposition of a single GO-NTA layer onto the grid via Langmuir-Schaefer transfer from IPA/H_2O.

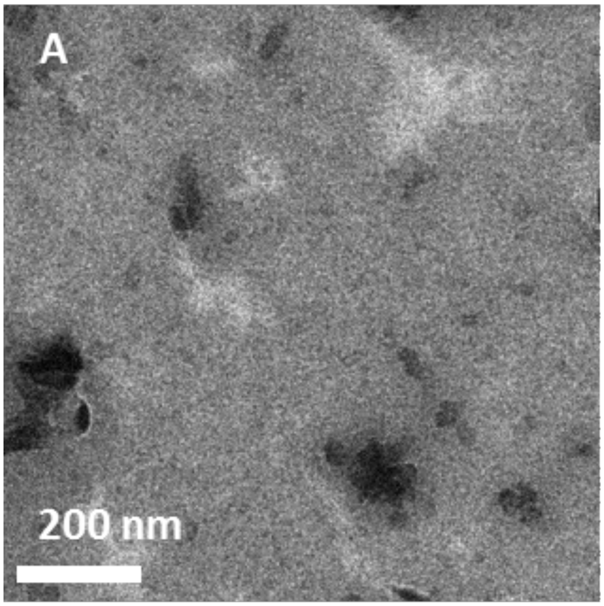

Figure S4. Negative stain TEM of his$_6$-T7 lysate deposited onto BSA-PABA-GO-NTA in the absence of Ni^{2+} activation.
